# Head Injuries

**Published:** 1895-02-09

**Authors:** 


### MEDICAL PROGRESS AND HOSPITAL CLINICS.

[*The Editor will be glad to receive offers of co-operation and contributions from members of the profession. All letters should be addressed to* THE EDITOR, THE LODGE, PORCHESTER SQUARE, LONDON, W.]

### HEAD INJURIES.

AN injury to the head is one of those things which must never be lightly considered, for even when of the most trivial nature it may have far-reaching consequences.

*Concussion of the brain* is one of the commonest results of a fall on to the head. A person falls and becomes unconscious, remains so for a short time, and recovers with a severe headache or, perhaps, with merely a dazed feeling. If the injury has been rather more severe, there will be slow pulse, the pupils will be dilated, contracted, or unequal, the respiration irregular, and the temperature low. The amount of reaction which follows corresponds to that of the depression. The most important point about these cases is to remember that a shaken brain means a bruised brain, with extravasations of blood, of greater or less amount, into its substance, on its surface, or into the spaces between its membranes. Any such injury may become inflamed, and suppuration may follow. If not relieved, this will inevitably end in death. If the inflammation do not pass on to suppuration, and if recovery take place, the powers of the brain will often be enfeebled in one or other direction. Too great care, cannot, therefore, be exercised in respect to a shaken brain.

The treatment of concussion is to keep the patient as quiet as possible during the stages of depression and reaction; during the former doing as little as possible, during the latter providing against excessive reaction by the application of ice-bags, or, at the least, raising the head; in any case keeping the patient on low diet until all signs of reaction have passed away, and then gradually bringing him back to ordinary diet. In all cases stimulants (beef-tea as well as alcohol) must be avoided, and the patient should be cautioned against the use of alcohol even in the future, a small amount of it often making such a person perfectly maniacal.

*Laceration of the scalp.* If the bone be exposed by the injury, or if subsequent inflammation spread to it, there is considerable risk of its attacking the membranes of the brain, or an abscess may form inside the skull, or the inflammation of the bone may become chronic, and, the skull becoming thickened, the bony prominence on the inside may press on the brain and cause epileptic fits or other disturbance of its functions. The most serious danger is inflammation of the cellular structure of the bone, and of its underlying dura mater. This is known by puffiness of the tissues near the wound, accompanied by fever, headache, intolerance of light, and other head symptoms. In these cases the probability is that an abscess or other collection of inflammatory exudation is taking place within the skull, and that by its pressure will cause coma, or by its poisonous effects may induce pyæmia and death.

*Fractures of the skull* are divided into those which affect the vertex, or top of the head, and those which affect the base of the skull. The former are by far the less dangerous, in that less vitally important nerve structures are in contact with the bones in that region, and that injury to the base of the skull is very apt to become septic from its involving parts communicating with the outer air. Fractures of the base usually injure one particular part of the skull, that known as the middle fossa, and hence the signs of such an injury are shown, in most cases, by indications of injury to the structures in that fossa, and of leakage of the contents of the skull (blood, cerebro-spinal fluid, brain substance) through the openings leading from the outside of the head in its direction, as in many cases one or more of these are affected.

Fractures of the vault are usually caused by direct injury. If this be produced by a pointed body so that a punctured wound results, immediate operation will be required, for not only must the splinters which have been driven down into the brain be removed, but drainage must be provided for the suppuration caused by the septic matters introduced.

Sometimes a large artery is divided by the broken bone and hæmorrhage takes place within the skull. This is shown by the patient more or less recovering consciousness after the accident and then relapsing into unconsciousness, with signs of pressure on the brain, such as stertorous respiration, unequal pupils, slow pulse, with unilateral paralysis, retention of urine, incontinence of fæces, &c. The treatment of such cases lies in trephining and ligaturing the divided artery. The advance in antiseptic knowledge has made manipulation of the contents of the skull very much more successful than it used to be, and opening the skull, like abdominal section, has few of the septic dangers it used to have, if done with proper precautions.

Fracture implicating the base is shown by blood extravasation under the conjunctiva, or into the eyelids, or cellular tissues of the back, or of the mastoid region of the skull. Or there may be bleeding from the nose or ear, or flow from these organs of a saline, pale fluid containing very little albumen; but, to be a sign of any importance, the flow of such fluids should last for some hours at the least. This indicates fracture of the base implicating the respective parts. In such cases the treatment lies in the antiseptic management of the wound, and especially of the external openings, such as the ear and nose, in keeping the reaction fever as low as possible, giving low diet, avoiding stimulants, and applying ice to the head, and in relieving any symptoms of pressure. Recovery in these cases is slow and uncertain, and will often leave mental or physical deficiencies of a more or less marked nature. The principal hope lies in the maintenance of complete asepticity.

*Inflammation of the brain following injury.* In the acute affections which may follow any head injury the symptoms are those of fever, vomiting, photophobia, headache, delirium, or convulsions, with those of pressure from the inevitable outpouring of fluid.

In the chronic affections the symptoms are less marked and come on slowly. There may be constant headache, passing on to mental symptoms of a more or less marked type, inequality of the pupils, and probably some amount of thickening at the site of the original injury.

In the acute form the treatment is that indicated under fracture, the later symptoms of that form of injury being principally those of inflammation of the membranes.

For chronic inflammation the usual medicinal treatment should be tried, and if the case gets worse, and the symptoms point definitely to any particular part of the brain, trephining may be done at this point.

Compression of the brain has been alluded to under the head of fractures, and it is necessary here to remark on the necessity which exists for noting any, even the smallest sign, of returning consciousness in a patient suffering from concussion; a sigh, a movement of the arm, may be the only indication that the stage of concussion has passed off, and that of reaction, with its dangers of hæmorrhage, &c., has come on; and if this sign has been missed, the patient may die of compression where the diagnosis had been concussion. The symptoms of compression from depressed bone follow immediately on those of concussion, no time intervening between them, whereas in the case of hæmorrhage there is nearly always an interval of more or less complete consciousness between the signs of concussion and the consecutive symptoms of compression.

